# Neural Marker of Habituation at 5 Months of Age Associated with Deferred Imitation Performance at 12 Months: A Longitudinal Study in the UK and The Gambia

**DOI:** 10.3390/children9070988

**Published:** 2022-07-01

**Authors:** Laura Katus, Bosiljka Milosavljevic, Maria Rozhko, Samantha McCann, Luke Mason, Ebrima Mbye, Ebou Touray, Sophie E. Moore, Clare E. Elwell, Sarah Lloyd-Fox, Michelle de Haan, The BRIGHT Study Team

**Affiliations:** 1 Centre for Family Research, University of Cambridge, Cambridge CB2 3RQ, UK; 2 Department of Psychology, University of Cambridge, Cambridge CB2 3EB, UK; bm618@cam.ac.uk (B.M.); sl868@cam.ac.uk (S.L.-F.); 3 Centre for Brain and Cognitive Development, Birkbeck College, London WC1E 7HX, UK; l.mason@bbk.ac.uk; 4 Psychology, School of Social Sciences, Nanyang Technological University (NTU), Singapore 639798, Singapore; rozhkomaria@gmail.com; 5 Department of Women and Children’s Health, Kings College London, London SE1 7EH, UK; samantha.mccann@kcl.ac.uk (S.M.); sophie.moore@kcl.ac.uk (S.E.M.); 6 Medical Research Council Unit The Gambia at the London School of Hygiene and Tropical Medicine, Keneba P.O. Box 273, The Gambia; embye@mrc.gm (E.M.); ebtouray@mrc.gm (E.T.); 7 Department of Medical Physics and Biomedical Engineering, University College London, London WC1E 6BT, UK; c.elwell@ucl.ac.uk; 8 Great Ormond Street Institute of Child Health, University College London, London WC1N 1EH, UK; m.de-haan@ucl.ac.uk; 9 Great Ormond Street Hospital for Children NHS Foundation Trust, London WC1N 1EH, UK

**Keywords:** habituation, novelty detection, event-related potentials, deferred imitation, cross-cultural

## Abstract

Across cultures, imitation provides a crucial route to learning during infancy. However, neural predictors which would enable early identification of infants at risk of suboptimal developmental outcomes are still rare. In this paper, we examine associations between ERP markers of habituation and novelty detection measured at 1 and 5 months of infant age in the UK (*n* = 61) and rural Gambia (*n* = 214) and infants’ responses on a deferred imitation task at 8 and 12 months. In both cohorts, habituation responses at 5 months significantly predicted deferred imitation responses at 12 months of age in both cohorts. Furthermore, ERP habituation responses explained a unique proportion of variance in deferred imitation scores which could not be accounted for by a neurobehavioural measure (Mullen Scales of Early Learning) conducted at 5 months of age. Our findings highlight the potential for ERP markers of habituation and novelty detection measured before 6 months of age to provide insight into later imitation abilities and memory development across diverse settings.

## 1. Introduction

A significant proportion of human learning is facilitated through imitation and observational learning [1]. Instead of trial-and-error learning, imitation of a proficient social partner represents a more efficient (though potentially more error-prone) way to acquire new skills. Across species, imitation plays a significant role throughout the lifespan, but bears particular importance during infancy: for example, presented with a novel action, young macaques and chimpanzees have been shown to be more likely than older individuals to reproduce an observed behaviour [2,3]. In human infants, the natural tendency to imitate behaviours can be utilised to draw conclusions not only about their ability to imitate, but also their symbolic thought and ability to mentally represent an action [4]. In contrast to elicited imitation, in which infants immediately copy a modelled action, deferred imitation paradigms introduce a delay between observation and imitation, requiring not only successful encoding, but also retention and retrieval of the action over the delay. Deferred imitation paradigms therefore provide a potential window into infants’ early memory development [5]. While imitation represents a crucial route to learning, the predictors of infants’ abilities to engage in this behaviour are not yet fully understood. There is some indication that neural markers tapping early memory development show concurrent associations with infants’ imitation behaviour [6], however, longitudinal associations of this link that would enable identification of infants who may be at risk of having suboptimal memory development early on are rare. Furthermore, studies examining the development and possible predictors of imitation behaviours suffer from a geographical bias, with most studies focusing on infants in Europe or North America. Addressing these twin gaps, this paper examines longitudinal associations of early neural markers with deferred imitation behaviour across two diverse cohorts in the UK and The Gambia, West Africa. Only by examining mechanistic associations across diverse settings can we draw inferences about the generalisability of mechanistic associations across contexts.

### 1.1. Development of Deferred Imitation Abilities across Infancy

Since Piaget’s first observations of infant imitation, proposing a sudden onset of imitation around 18–24 months of age (e.g., [4]), it has been shown that even younger infants [7,8] and indeed newborns [9] show gradual improvements in their ability to copy salient actions. The deferred imitation paradigm provides a formalised test of infants’ ability to imitate: typically, infants are presented with a range of novel objects, for which an experimenter models a target action. Infants are then either allowed to imitate directly following this demonstration (immediate imitation) or following a delay (deferred imitation). The most dramatic developmental changes can be observed across infancy with regard to (a) the delay tolerated between demonstration and imitation, (b) the number of demonstrations necessary for successful encoding, (c) the degree to which infants can transfer learned actions to novel objects and settings and (d) the degree to which imitation hinges on the interconnectedness of steps (e.g., whether sequences are arbitrary or causal/enabling in nature). As reviewed in Jones and Herbert [10], around 25% of six-month-olds [11] could reproduce a novel action after a 24 h delay, increasing to 50% by nine months of age [12]. At 14 months, the majority (77%) of infants could retain an action sequence over a one-week delay [13]. Furthermore, infants at six months were, for the most part, unable to succeed even when presented with an action six times, whereas older infants (nine months and above) successfully imitated after only one to three demonstrations [10]. Feature changes (e.g., using a differently coloured object in the test compared to demonstration phase), as well as changes to the testing environment (e.g., being assessed in a laboratory setting vs. in the home) also affect infants to a greater degree at younger ages. At six months of age, infants’ imitation behaviour was shown to be reduced when a change in the presentation context had occurred [14]. This rigidity is typical for younger infants and is gradually replaced by a change towards higher representational flexibility as infants develop. While six-month-old infants could still successfully imitate when changing rooms within their home for the demonstration and test phase [15], they could not cope with a transfer from the laboratory setting to the home [14]. From 12 months, infants were able to imitate after one-week delay regardless of context changes [16,17]. As for object feature changes, six-month-olds were able to imitate actions after a 24 h delay when tested with the original object and no contextual changes to the testing environment [18,19]. From 12 months, infants could still imitate if the colour of an object changed, but not if its form changed, whereas by 18 months they were able to generalise across both dimensions [19]. Across ages, enabling sequences (in which the order of steps is contingent on one another, e.g., building a rattle by placing a ball in a cup and closing the lid) are more frequently imitated than arbitrary ones (in which steps can be performed in any order), [18,20,21,22] as the interconnectedness of steps acts as a cue to recall the following step. In studying 24-month-old infants, Bauer et al. [23,24,25] have shown that enabling sequences are more reliably imitated by participants both immediately after demonstration and following a delay, independently of number of steps, number of presentations, or length of delay. In sum, from around six months of age infants are able to engage in basic forms of object-based imitation, with great developmental gains thereafter.

### 1.2. Imitation Behaviours across Cultures

Imitation is a universal means of learning and several studies have demonstrated commonalities across cultures. For example, similar developmental levels of spontaneous imitation have been observed for toddlers from the United States and those from Papua New Guinea, even though the imitative play they engaged in was found to be centred on themes relevant to their social environments [26]. Imitation of task-irrelevant features or over-imitation (in which infants do not only copy those actions necessary to achieve a sequence’s end-state, but also irrelevant actions) has been shown to occur to similar degrees in both urban Australian and children from the Kalahari San population in southern Africa [27]. Furthermore, Goertz et al. [28] found no difference in either baseline performance of the target action or imitation between German and Cameroonian infants from the Nso community. However, it has been argued that cultural differences may increase with age: Graf et al. [29] demonstrated that at nine months, but not at six months, German infants had overall higher scores in the baseline and imitation phase than the Cameroonian infants. This may in part be attributed to familiarity with toy play, as well as the ecological validity of the assessment situation: Teiser and colleagues [30] examined whether age of the demonstrator (child or adult) differed across the two cultures, since in the Cameroonian culture more child–infant interaction is prevalent, whereas in German culture adult–infant interaction is more common. At nine months of age, all infants, regardless of cultural group imitated more with an adult than with a child model. Furthermore, German infants performed significantly higher numbers of target actions at both six and nine months in the test compared to the baseline phase while this was only true for the Cameroonian infants at nine months. Another line of research suggesting that cultural differences may take effect at older age points shows that at 5–10 years of age, Mayan children were more likely than their European–American counterparts to attend to novel actions not directly involving them [31]. This indicates that culture as well as the introduction of formal schooling may elicit different attentional styles across settings. In sum, these studies show that imitation can be elicited across cultures, regardless of whether toy play is the norm or the exception and that object-based imitation shows developmental gains with age. While frequency of imitation may differ across settings, an investigation of individual differences in relation to early-life predictors could add important insights: firstly, early predictors may inform our understanding of mechanistic associations across development, which can help us understand the reasons for performance differences across populations. Secondly, a better understanding of longitudinal associations can support early identification of those infants who may be developing atypically. In this context, particularly early neural markers may play an important role, since they can unobtrusively be collected in young infants even before their motor repertoire allows for meaningful behavioural assessments. For example, assessment such as the Mullen Scales of Early Learning (MSEL) are limited to a small number of items below six months of age, limiting its ability to detect meaningful variance. Some evidence from at-risk cohorts indicates that on a behavioural level, differences may only become apparent from six months of age at the earliest [32], and that behavioural measures become better at detecting developmental delays with increasing age [33]. An investigation of such neural markers and their association with longitudinal behavioural development is therefore warranted.

### 1.3. Neural Markers Associated with Deferred Imitation

Even before infant imitation behaviour can be reliably assessed, neural markers (e.g., based on electroencephalography [EEG] and, specifically, event-related potentials [ERP]) of the contributing processes, such as infants’ ability to encode and retain stimuli can provide insight into infant memory development. Individual differences in these neural markers have been shown to be associated with concurrent imitation behaviours. Bauer et al. [34] found that nine-month-old infants presented with a range of action sequences showed differential ERP responses to still images of actions they had observed vs. novel actions after a one-week delay. Developmentally, Bauer and colleagues [35] also showed that while both nine- and ten-month olds showed ERP differences to novel vs. familiar action sequences immediately following observation (indexing successful encoding), this process was more robust in ten-month olds. Furthermore, only ten-, but not nine-month-olds showed ERP differences after a one-month delay, indicating that only the older infants were able to recall the observed actions. Further highlighting differences between encoding and retrieval, Morasch and Bell [36] showed that while infants who could perform ordered recall of a deferred imitation sequence after 24 h showed no difference in 6–9 Hz EEG power during the initial demonstration of action sequences compared to those infants who could not. Only those infants who could successfully imitate after a 24 h delay showed an increase in 6–9 Hz EEG power at anterior and temporal locations between the baseline and task condition. Examining infants’ ERP response to novel vs. familiar stimuli, Heimann et al. [6] found that greater negativity in the negative central (Nc) ERP component in 14–15-month-old infants was linked to higher rates of deferred imitation. Furthermore, in a related study Nordquist et al. [37], report a similar association between ERP novelty and deferred imitation, which they attribute to the domain-specificity of the ERP markers for early explicit memory development. Findings suggesting a link of neural markers of habituation and novelty detection with concurrent deferred imitation behaviours call for an examination of the longitudinal stability of such associations.

### 1.4. Cross-Cultural Development of Early Neural Markers of Habituation and Novelty Detection

Our previous work examining neural markers of habituation and novelty detection across two cohorts in the UK and The Gambia has shown a protracted developmental trajectory of robust novelty and habituation responses in the Gambian compared to the UK cohort [38,39]. In an fNIRS paradigm assessing infants at 5 and 8 months, Lloyd-Fox et al. [39] showed that, in the UK cohort, both habituation and novelty responses were evident at 5 months and became more robust from 5 to 8 months of age. However, a different pattern emerged in The Gambia, with infants requiring a greater number of trials to show the same degree of neural response suppression, and not showing a response recovery when presented with novel stimuli at either 5 or 8 months of age. Similarly, Katus et al. [38] showed that on an EEG paradigm of habituation and novelty detection, infants in both the UK and The Gambia showed immature novelty habituation and novelty responses at 1 months of age. However, from 1 to 5 months of age, infants in the UK cohort showed an increase in these responses, whereas at a group-level, no robust habituation and novelty detection was observed in the Gambian cohort. These differences in group-level developmental trajectories require further contextualisation, for example by investigating whether individual differences across assessment modalities show stability over time, and are longitudinally associated with developmental outcomes.

Building on this background, this paper is the first to describe longitudinal associations between the ERP markers of habituation and novelty detection collected at 1 and 5 months of age, and the deferred imitation scores at 8 and 12 months of age. We hereby aim to assess this link in both the UK and The Gambia, to answer a common set of questions: through an analysis of such mechanistic associations in the UK cohort, we aim to expand the current literature from other high-income study sites cohorts by examining whether reported brain-behaviour associations [6,37] hold longitudinally. By including the Gambian cohort, we then aim to assess whether such mechanistic associations may have a degree of universality, in that they are also present in previously understudied infant population, with different environmental exposures and cultural practices. An investigation of the predictive utility of early neural markers for longitudinal developmental outcomes represents the first step in understanding whether neural markers show utility in contributing to early identification and ultimately intervention. Neural markers may also show stronger associations with developmental outcomes than behavioural measures collected at early age points, which may make them particularly useful to implement with young infants. Furthermore, to assess the specificity of this association, we will examine whether ERP markers account for variance in imitation scores that goes above and beyond what can be explained by general neurodevelopmental scores assessed by the MSEL at five months of age. In addition to contributing to the knowledge-base on domain-specific association of early ERP markers and performance deferred imitation tasks, this also allows us to better understand the utility of employing neuroimaging in addition to neurobehavioural assessments across contexts.

### 1.5. Aims and Hypotheses

The current study was conducted as part of the Brain Imaging for Global Health project (BRIGHT project, https://www.globalfnirs.org/the-bright-project/, accessed on 30 June 2022) which longitudinally assessed two parallel infant cohorts in the UK (*n* = 61) and The Gambia (*n* = 214), with longitudinal visits at 7–14 days, 1, 5, 8, 12, 18 and 24 months of infant age. The protocol incorporated neuroimaging (functional near infrared spectroscopy [fNIRS], EEG) and eye tracking alongside behavioural measures with the MSEL performed at 5, 8, 12, 18 and 24 months of infant age. The EEG assessments were run at 1, 5 and 18 months; here, data from the 1- and 5-month age point are considered to enable prediction of deferred imitation scores gathered at 8 and 12 months of age. In the current analysis, we examine associations between the 5-month age point of the MSEL, the EEG responses at 1 and 5 months, and deferred imitation scores at 8 and 12 months of age. These associations were examined separately for the UK and the Gambian cohort to find out whether some mechanistic associations generalise across these two diverse settings, indicating a degree of universality. We hypothesise that:

Infants will show an increase in their deferred imitation abilities between 8 and 12 months.

Neural markers of habituation and novelty detection at 1 and 5 months of age will show longitudinal associations with deferred imitation scores at 8 and 12 months of age.

Neural markers will account for a unique proportion of variance in deferred imitation scores, that goes over and above variance accounted for by MSEL scores.

## 2. Method

### 2.1. Participants

In the UK, families were recruited at their 32–36 weeks’ clinic visit to the Rosie Hospital, Cambridge University Hospitals NHS Foundation Trust. In The Gambia, families were recruited during the second and third trimester of gestation via antenatal clinic visits to the Keneba field station of the Medical Research Council Unit The Gambia at the London School of Hygiene and Tropical Medicine (MRCG at LSHTM). At both sites, participants were eligible if (1) mothers were above 18 years of age, (2) living primarily in the study’s catchment area, (3) infants were born at term (i.e., 37–42 weeks gestation) and (4) infants were not identified with neurological deficits during neonatal checks. In the UK, only infants with a birthweight of >2.5 kg were enrolled. Furthermore, in The Gambia only predominantly Mandinka speaking families were recruited, who represent the majority ethnic group in the region data collection took place, to avoid confounds of translating and adapting language-based measures into multiple languages. No restrictions were made on infants’ birthweight in this cohort, because multiple environmental factors (undernutrition, frequent infections) prevalent in this region frequently lead to growth faltering with regard to both infants’ length and weight over the first months of life [40]. Furthermore, because one of the aims of the BRIGHT project was to study the consequences of growth faltering on brain development, infants were retained in analyses.

### 2.2. ERP Study

The method for this study is described in detail in Katus et al. [38]. EEG measures were obtained at 1-, 5-months of infant age. Infants were presented with a total of 1000 auditory stimuli (100 ms duration, 5 ms ramp up and down time, inter-stimulus interval 650–750 ms, mean length 700 ms) of three different categories: *Frequent* sounds (500 Hz pure tones, presented at 80% probability), *Infrequent* sounds (white noise, presented at 10% probability) and *Trial Unique* sounds (presented at 10% probability). *Trial Unique* stimuli consisted of a range of different sounds, such as clicks, tones, digitized vocalizations and syllables (adapted from [41]) and were each only presented once. Stimuli were presented through wireless Sony TMR-RF810R headphones, at 60dB SPL.

#### 2.2.1. Procedure

Data were recorded via a Neuroelectrics Enobio8 system, Neurolectrics, Barcelona, Spain) with eight electrodes placed at Fz, FC1/2, C1/z/2 and CP1/2. Data were recorded in reference to infants’ left mastoid and this reference was retained throughout data analysis. At the 1-month age point, infants were asleep during data acquisition, whereas at 5 months infants were assessed while awake and quietly interacting with one of the researchers. As discussed in Katus et al. [38], this did not result in a measurable difference in P3 mean amplitudes.

#### 2.2.2. Pre-Processing

Data were pre-processed via customised Matlab routines (Mathworks, Inc., 2015, Natick, MA, USA). A bandpass filter of 0.5–30 Hz was applied (blackman, filter order 5500), data were then offset corrected for a 32 ms timing delay and segmented from 200 ms pre- to 800 ms post-stimulus onset. Artifacts were removed via a simple voltage threshold of ±100 µV. Flatlining epochs with a change of less than 0.1 µV in an epoch were also rejected. The condition with fewest valid trials was identified and a random sample of the same size was chosen from both other conditions. Datasets with <15 trials per condition were discarded. To enable the extraction of habituation markers, data sets with <45 Frequent stimuli were discarded.

Scoring and outcome variables. As a measure of novelty detection, we calculated the difference between P3 mean amplitudes to Trial Unique and Frequent sounds, which was normalised for individual variance in overall ERP amplitudes by dividing responses by amplitudes to Frequent sounds (Trial Unique—Frequent/Frequent). As a measure of habituation, we examined the difference in responses to trials 1–15 and 30–45, normalised by responses to the first 15 trials (Trial 1–15—Trial 31–45/Trial 1–15, for details see Katus et al. under rev).

### 2.3. Deferred Imitation

**Figure 1:**
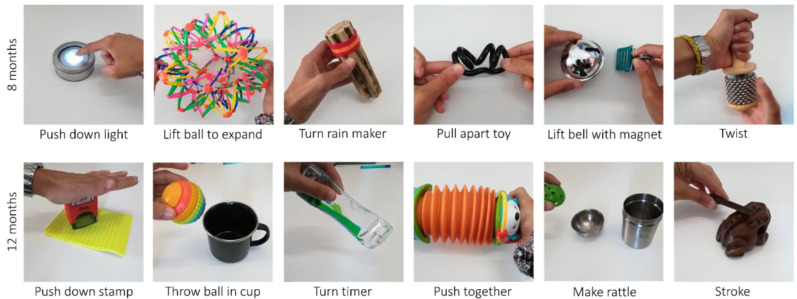
Objects used to demonstrate and elicit novel actions in deferred imitation task at 8 months (top row) and 12 months (bottom row). Copyright: Laura Katus.

Infants completed the deferred imitation task at their 8- and 12-month visits. Stimuli for this study were designed to be (1) motorically manageable for young infants, (2) novel to both infants in the UK and The Gambia and (3) independent of whole-body movements, as fNIRS data were recorded during this task in the UK cohort. Here, we examine only the behavioural data. Items and target actions are illustrated in Figure 1.

#### 2.3.1. Procedure

**Figure 2:**
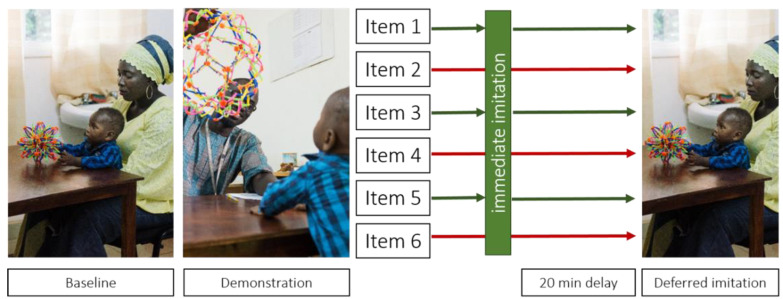
Procedure of the deferred imitation task. During the baseline phase, infants were allowed to explore the toy and it was recorded whether or not they performed the target action during this phase. In the demonstration phase, the experimenter modelled the action three times. Half of the items were then given back to the infant immediately after demonstration (immediate imitation phase). After a 20 min delay infants were presented with all six items (deferred imitation phase). Which half of the items was included in the immediate imitation phase was counterbalanced across infants. Photo panels reprinted with permission of Ian Farrell (2022).

Infants sat on their parents’ lap at a table facing the experimenter. First, infants were presented with an item and were given time to freely explore it (*baseline* phase). After 30 s, the experimenter demonstrated the target action three times (*demonstration* phase). This procedure was repeated for all six items. For half of the items, the demonstration phase was followed by an *immediate imitation* phase, during which the experimenter presented the infant with the item directly following the demonstration for this item. Across participants, it was counterbalanced which half of items were presented during the immediate imitation phase. After a 20 min delay, infants were in turn presented with each item and their performance of the target action was recorded. The procedure is illustrated in Figure 2.

#### 2.3.2. Task Design and Reliability Assessments

Items were designed to take into consideration differences between sites regarding prior exposure to objects, as well as limited motor abilities of infants below one year of age. Items were aimed at being slightly more demanding at the 12-month age point, to prevent ceiling effects. Administrators of this paradigm had previous experience with standardised behavioural infant testing from other studies on the BRIGHT project (e.g., the MSEL). Training on this task took the form of a detailed protocol description, one in-person training and several regular check-up meetings to discuss questions and provide feedback on specific items. As part of these discussions, scoring criteria were simplified and made more objective, leading to high inter-rater reliability rates (post hoc video-based scoring yielded ICC’s > 0.8).

#### 2.3.3. Scoring and Outcome Variables

Our scoring procedures followed common principles applied in deferred studies (c.f., Jones and Herbert, 2006). The majority of actions had only one step and were scored as 0 (not completed) or 1 (completed). For some items, two steps were scored (i.e., picked up object with one hand either side = 1, pulled object apart = 2). Responses during the baseline and imitation phases were all scored according to the number of steps completed. Infants’ scores during the baseline phase were then subtracted from those in the immediate and the deferred phase, obtaining an imitation score that disregarded the number of target actions completed prior to demonstration.

### 2.4. Mullen Scales of Early Learning (MSEL)

The MSEL was performed at the 5-, 8-, 12-, 18-, and 24-month visits of the BRIGHT project. Here, data from the 5-month age point are presented. The administration of the MSEL as well as the adaptations made for use in the Gambian context are described in Milosavljevic et al. [42]. The MSEL [43] comprises five subscales, measuring fine motor (e.g., picking up a small object), gross motor (e.g., sitting with arms free), expressive (e.g., producing syllable strings) and receptive (e.g., orienting to sound) language development, as well as infants’ visual reception (e.g., attending to moving visual target). The scale contains items for infants and children aged 0–68 months, which are presented in increasing order of difficulty. Administration begins by establishing a baseline of three successfully completed items in a row, and continues until a child is unable to complete three consecutive items. Raw scores can then be used to obtain age-normed t-scores (M = 50, SD = 10). All scales with exception of the gross motor scale can be used to obtain a composite t-score (M = 100, SD = 15). Previous analyses by our group [42] have highlighted good internal consistency (r = 0.75–0.83) and test–retest reliability (0.78–0.96) across subscales.

#### MSEL Scoring and Outcome Variables

Based on prior work using both a verbal and non-verbal outcome measure on basis of the MSEL [44], we obtained sum scores for infants’ verbal development (VDQ, based on the expressive and receptive language subscales) and non-verbal development (PDQ, based on the fine motor and visual reception subscales). This distinction was made to possibly disentangle the differential impact of early perceptual and fine motor skills (assessed by the PDQ) and verbal, declarative processes (assessed by the VDQ) for later imitation.

### 2.5. Statistical Analyses

For the deferred imitation task, baseline corrected scores were modelled in one repeated measures ANOVA per site by Condition (immediate/deferred) and Age (8 months/12 months), followed by appropriate post hoc comparisons. For the ERP task, a detailed component analysis is reported in Katus et al. [38]. Here, we expanded on our previous analyses by examining associations between our ERP novelty and habituation markers measured at 1 and 5 months with the deferred imitation scores at 8 and 12 months of age. We first examined the correlation between the ERP markers and deferred imitation scores at 8 and 12 months. Subsequently, hierarchical regression models were used to examine whether variance accounted for in deferred imitation scores at 8 and 12 months increased when adding ERP markers at 1 and 5 months. Hereby, MSEL scores at 5 months (verbal development quotient [VDQ] and non-verbal development quotient [PDQ]) were entered to the baseline model in a first step. ERP responses were entered in a second step. This procedure was followed in separate models for each site, in order to examine the potential generalisability of mechanistic associations across contexts.

## 3. Results

### 3.1. Data Rejection and Sample Characteristics

Sample characteristics for infants included and excluded in the analyses can be found in Table 1 and Table 2. No significant differences were observed between infants that did and did not contribute data for the deferred imitation task at 12 months.

**Figure 3:**
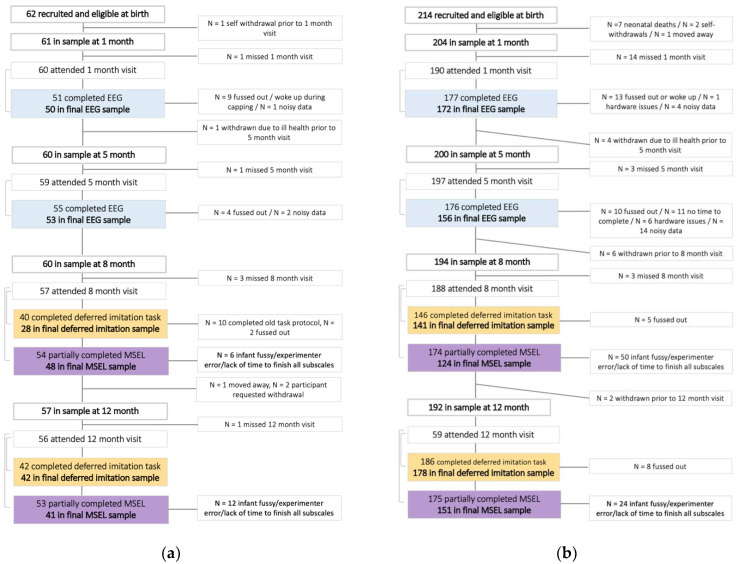
Numbers of infants retained in and rejected from analyses and reasons for exclusions in the UK cohort (**a**) and the Gambian cohort (**b**). Final numbers included are highlighted for the EEG task (blue), the deferred imitation task (yellow) and the MSEL (purple).

Data rejection/retention rates for both cohorts can be found in Figure 3. As can be seen, the reasons for missing data included (1) infants missing the visit, (2) infants being too fussy to be assessed, and (3) lack of time on the testing day. Furthermore, ERP data were rejected when fewer than 15 artifact-free trials per condition were retained after pre-processing. For the deferred imitation task, data were also rejected when the delay between the immediate and the deferred phases was <10 min or >45 min, or fewer than three trials could be administered. For the MSEL, data could not be included where experimenter error occurred on one of several subscales, preventing the generation of an overall sum score from being obtained.

### 3.2. Deferred Imitation Behavioural Results

**Figure 4:**
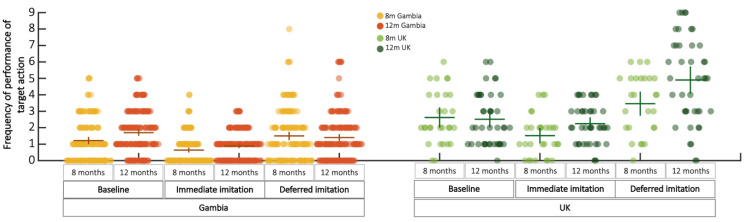
Frequency at which infants performed target action per condition, age point and site for the deferred imitation task.

Raw scores for the baseline, immediate and deferred condition of the deferred imitation task are visualised in Figure 4 and summarized in Table 3. Raw scores were baseline corrected by subtracting infants’ scores during baseline from their score in the immediate and deferred condition and these corrected scores were used in all further analyses, to control for spontaneous production of target behaviours.

Deferred imitation scores were modelled in a repeated measures ANOVA with within factors Condition (immediate/deferred) and Age (8 month/12 month). In the UK cohort, a main effect was found for Condition (*F*_1,15_ = 23.388, *p* < 0.001, *η_p_*^2^ = 0.609) but not Age (*F*_1,15_ = 1.5, *p* = 0.240, *η_p_*^2^ = 0.091), and no interaction was observed (*F_1,15_* = 1.097, *p* = 0.311, *η_p_*^2^ = 0.068). The condition effect was driven by higher scores in the deferred compared to the immediate condition at both the 8-month (*t*_25_ = −3.521, *p* = 0.002, *d* = 0.691) and 12-month (*t*_40_ = −7.111, *p* < 0.001, *d* = 1.11) age point. In The Gambian cohort, we found main effects for Condition (*F*_1,126_ = 57.458, *p* < 0.001, *η_p_*^2^ = 0.313) and Age (*F*_1,126_ = 12.226, *p* = 0.001, *η_p_*^2^ = 0.088), but no interaction effect (*F*_1,126_ = 2.996, *p* = 0.086, *η_p_*^2^ = 0.023). Post hoc tests showed that main effects were driven by higher scores in the deferred compared to the immediate condition at the 8 month (*t*_136_ = −3.457, *p* = 0.001, *d* = −0.295) and the 12-month (*t*_178_ = −9.623, *p* < 0.001, *d* = −0.719) age point, and that scores were higher at 12 months compared to 8 months in both the immediate (*t*_131_ = −2.563, *p* = 0.012, *d* = −0.223) and the deferred imitation condition (*t*_127_ = −4.177, *p* < 0.001, *d* = −0.369).

To help contextualise these findings, exploratory analyses were conducted: while items were designed to be appropriate to the motor abilities to infants at each age point, we examined whether older infants found it easier to handle the presented objects and perform target actions. To assess this, we compared baseline scores across the age points. We found no evidence for differences in baseline scores between the 8- and 12-month age point in either the UK cohort (*t*_18_ = −0.725, *p* = 0.478, *d* = −0.166) or the Gambian cohort (*t*_133_ = 1.096, *p* = 0.275, *d* = 0.095). To assess whether these non-significant results presented evidence for a true null effect, we calculated the Bayes Factor testing the likelihood of the current data being obtained under the null compared to the alternative hypothesis (*BF*_01_) and found it to be high for both the UK (*BF*_01_ = 4.179) and the Gambian (*BF*_01_ = 3.616) cohort. A *BF* > 3, indicates that the observed data is at least three times more likely under the null- than under the alternative, and is generally accepted as indicating reliable evidence for the null hypothesis to be true [45]. Furthermore, since infants in The Gambia were overall less familiar with table-top toy play than the infants in the UK, we also assessed if the engagement with the items differed between cohorts. To this end, we assigned a score of either 0 (did not touch) or 1 (touched) for each item the infant was presented with. Comparing these scores across sites per age point, we found that on group level, infants’ engagement with the items did not differ at either 8 months (*t*_46_ = −0.302, *p* = 0.764, *BF*_01_ = 3.766) or 12 months (*t*_62_ = −1.101, *p* = 275, *BF*_01_ = 3.136).

**Figure 5:**
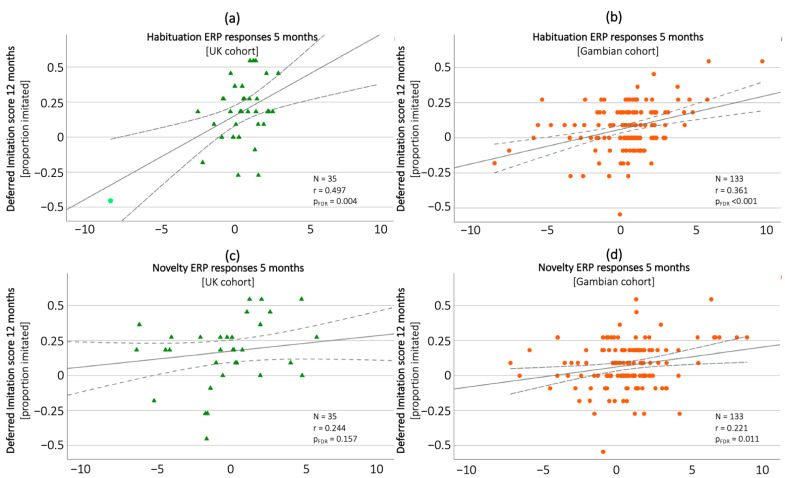
Correlations between ERP habituation (in UK (**a**) and The Gambia (**b**)) and novelty detection (in the UK (**c**) and The Gambia (**d**)) measures at 5 months and deferred imitation scores at 12 months.

To assess whether our two measures of infant memory development (ERP markers and deferred imitation scores) showed the anticipated associations at both sites, we examined correlations between the ERP habituation and novelty detection indices at 1 and 5 months, with deferred imitation scores at 8 and 12 months. Visual inspection revealed one outlying value in the UK cohort for the habituation response of the EEG (highlighted in Figure 5). Since the value was not out of range when compared to the bigger Gambian cohort it was retained. Preliminary data checks showed that the value did not change the effects described below for the correlation analyses. Results were corrected for multiple comparisons via FDR correction. We did not find any correlation between the ERP habituation and novelty detection indices at either 1 or 5 months with deferred imitation scores at 8 months in either the UK or the Gambian cohort (*p_FDR_* all > 0.264). In the UK cohort, we found a positive correlation between the ERP habituation index at 5 months and deferred imitation scores at 12 months (*r* = 0.497, *n* = 35, *p_FDR_* = 0.004). No such associations were found for the habituation scores at 1 month, or novelty detection scores at either age point. In the Gambian cohort, we found associations between the ERP habituation (*r* = 0.361, *n* = 133, *p_FDR_* < 0.001) and novelty detection (*r* = 0.221, *n* = 133, *p_FDR_* = 0.011) indices at 5 months, but not at 1 month, with deferred imitation scores at 12 months. Associations between deferred imitation at 12 months and ERP responses at 5 months are illustrated in Figure 5.

Since no correlations were found between the ERP markers and the deferred imitation scores at 8 months, we only proceeded to model the 12-month deferred imitation scores as an outcome for all further analyses. Hereby, we were interested to find out whether the ERP indices explained variance over and above what could be accounted for by a broader index of neurodevelopmental status, since ERP markers and deferred imitation scores were hypothesised to tap the same underlying domain of memory development. In hierarchical regression models (one per site), with 12-month deferred imitation scores as an outcome, we entered MSEL scores at 5 months to the baseline model, and then, in a second step entered ERP indices at 1 and 5 months of age. The variance accounted for increased with addition of these markers (*R*^2^*-change* UK = 38.5%, Gambia = 12.9%). In both cohorts, we found that MSEL scores were not significant as predictors of deferred imitation scores at 12 months of age (*p* all > 0.178). In both cohorts, ERP habituation markers at 5 months, but not at 1 month, were significant predictors (UK: *b* = 0.053, *t* = 2.207, *p* = 0.040; Gambia: *b* = 0.021, *t* = 2.336, *p* = 0.022). While the ERP novelty detection marker correlated significantly with deferred imitation scores at 12 months in the Gambian cohort, it was not found to be a significant predictor in the hierarchical regression model (Gambia: *b* = 0.007, *t* = 1.164, *p* = 0.247).

We conducted an additional exploratory analysis, to assess whether the fact that scores in the immediate imitation condition being reduced relative to the deferred imitation condition could index infants’ habituation to the presented objects over the baseline, demonstration and immediate imitation phase. We found that ERP habituation correlated significantly with Immediate imitation at 8 months in the Gambian cohort only (*r* = 0.248, *n* = 100, *p* = 0.006). This association was not found for the 12-month immediate imitation, and for neither age point in the UK cohort. In a hierarchical regression model, ERP habituation at 5 months was not a significant predictor of immediate imitation at 8 months, the association did not hold in this more stringent analysis.

## 4. Discussion

This study assessed infants’ deferred imitation development as well as associations with early neural markers across two diverse cohorts in the UK and The Gambia. While some prior evidence suggests a potential link between neural markers of habituation and novelty detection with concurrent deferred imitation abilities [6,37], longitudinal associations have thus far received only limited attention. Our study also extends knowledge gained from previous studies comparing imitation behaviour and its developmental change in across two diverse cohorts [28,29,30]. It is also the first to assess links between neural and behavioural measures of infant memory development across two diverse cohorts. Therefore, it holds the potential to assess whether mechanistic associations generalise across infants from different settings. We found that in both cohorts, the degree to which infants habituated to the ERP stimuli at 5 months of age was associated with their deferred imitation abilities at 12 months. No such associations were found for the degree to which they displayed novelty detection on the basis of their ERP at either one or five months. Lastly, ERP markers at one and five months were not predictive of deferred imitation at eight months of age.

### 4.1. Developmental Changes in Deferred Imitation and Association with ERP Markers

We found the hypothesised increase in deferred imitation scores in the Gambian cohort. While no such increase was found in the UK cohort, this may have been due to a limited sample size (see Strengths and limitations section, below). Moreover, counterintuitively, we observed more imitation behaviours in the deferred relative to the immediate condition in both the UK and the Gambian cohort at both 8 and 12 months of age. While this finding seems to be at odds with the differential memory demands placed on infants in these conditions, observations from assessments suggest that it can be attributed to infants’ decreasing interest in the presented objects over the baseline, demonstration, and immediate imitation phase. After the 20 min delay (in which infants switched to a screen-based passive task), they generally showed an increased engagement with the imitation task compared to the immediate imitation phase. This is in part corroborated by our exploratory analysis, showing a correlation between infants ERP habituation responses and immediate imitation scores at eight months of age in the Gambian cohort.

Overall, we only found evidence for our hypothesis that ERP markers would predict deferred imitation; while we did not find associations between the ERP markers and deferred imitation at 8 months at either site, we did find links with the ERP at 5 months and deferred imitation at 12 months. Several potential reasons could explain this. First, our outcome measures were quite stringently scored, by correcting deferred imitation scores for spontaneous performance of target actions during the baseline phase per infant (as is common practice in these kinds of tasks) [5,46]. Additionally, scores overall were low in comparison to the baseline at the eight-month age point. Indeed, while several studies highlight that infants show gradual improvements in their deferred imitation abilities across infancy, other studies (e.g., [35,46]) highlight that developmental gains are particularly notable around 9–12 months of age, where mastery of many crucial aspects (e.g., the ability for ordered recall, [5] of deferred imitation tasks manifests as an almost step-like change in infants’ performance. It therefore may be, that at eight months of age, the task was not able to pick up meaningful individual differences in early memory development leading to an absence in associations with other factors such as the ERP markers.

In a similar way, we found ERP markers at one month were not associated with deferred imitation at either eight or twelve months. In our previous work, we found that in both the UK cohort and The Gambian cohort, infants at one month did not yet show either novelty or habituation responses based on their ERP. This is in line with prior research suggesting that these robustly emerge by two months of age at the earliest [47,48]. It may therefore be, that not only are these responses absent at group level at one month of age, but also that individual differences are not yet meaningful as a measurement of early memory development.

### 4.2. Domain-Specific Links of Deferred Imitation with ERP Markers and MSEL Scales

While we hypothesised to see similar associations of ERP markers and deferred imitation scores across the domains of habituation and novelty detection due to the complementary nature of these processes, we found the most robust associations with infants’ habituation responses. In the UK, only ERP habituation responses were associated with deferred imitation at 12 months and provided a significant predictor in the regression models. In The Gambia, both habituation and novelty detection at 5 months were correlated with deferred imitation at 12 months, but only habituation responses significantly predicted variance over and above MSEL scores in the regression models. In this context, it needs to be noted that in our prior work using fNIRS we found habituation responses early on in development, whereas novelty detection only emerged at the 18-month age point [39]. It may therefore be, that habituation might emerge and be measurable earlier in development, and therefore serve as a more robust neural marker in young infants. Since other studies [6,37] report links with novelty detection in older children (14–15 months of age), it may also be that sensitivity of markers of these domains changes across development.

Even though a strong argument can be made for deferred imitation to tap the same cognitive resources as recall memory and verbal recall (e.g., on the basis of its developmental progression and underlying neural circuitry) [5,49] we did not find an association with the MSEL VDQ and deferred imitation in the current study. Furthermore, while the visual reception and fine motor scales contributing to the MSEL PDQ both share overlapping demands with deferred imitation tasks, we also did not find those scales to be a significant predictor. This finding may be attributable to MSEL showing increasing group-level and individual discrimination and therefore predictive utility with age: in our previous work in the context of assessing infants in rural Gambia, we found that most infants perform consistently with norm scores at 5–9 months of age but started to show increasing discrepancies (with lower scores than the age-norms) from 10–14 months of age. This again highlights that behavioural measures increase in their predictive validity with increasing age, but are limited during the first few months of development.

### 4.3. Strengths and Limitations

Firstly, sample characteristics need to be considered when interpreting these results. We have achieved the longitudinal collection of neurophysiological and behavioural data across two geographically and socio-demographically diverse cohorts. However, since longitudinal associations required contribution of data across several age points and assessment modalities, there was a marked reduction in sample size for the overall smaller UK cohort. Cohorts were designed to be different in size as the main goal of the BRIGHT project was to tease apart longitudinal developmental trajectories in The Gambia. Especially analyses examining changes across age points may therefore be underpowered, which might also have contributed to non-significant findings (e.g., the absence of a main effect for age in the deferred imitation task). While this may limit the robustness of the findings from the UK cohort, it is important to note that several prior studies [6,37] suffer from similarly limited sample sizes. The fact that our main findings align with these prior studies and also across cohorts within this study strengthens our confidence that we are not observing spurious effects.

Secondly, while items of this task were developed in parallel across sites, the overall reduced familiarity with toy play in the Gambian cohort and resulting situational novelty needs to be considered. While this paper was primarily interested in understanding mechanistic links between early ERP markers and deferred imitation without a direct site comparison, individual differences in the Gambian cohort may to a greater part reflect which infants were better able to cope with a novel situation than in the UK. However, our additional exploratory analyses allowed us to contextualise our findings in a way that highlights several commonalities across age points and study sites: first, we did not find site differences with regard to infants’ engagement with the presented objects. This is not to say that more subtle differences (e.g., infants total time handling the objects, or the latency between being presented with and first engaging with the object) do not differ across contexts, though such an analysis is beyond the aims of this specific study. Thirdly, while confounds with motor development may come into play, our analyses comparing baseline performance did not show systematic differences, and the lack of an association between the MSEL PDQ, which includes fine motor scores further corroborates this.

Lastly, performance of target action during the baseline performance was high. This was due to the need to design items easy enough to be motorically manageable for infants of this young age group, which for the most part resulted in one-step actions which infants some of the time would perform during baseline. Other studies (e.g., [46]) of infants below 12 months of age report that around 25–30% of target actions were performed during baseline, which is in line with findings in our study. While high baseline performance might make it harder to measure imitation over and above baseline performance, the fact that scores were controlled for this at least precludes erroneous attribution of behaviours to imitation.

## 5. Conclusions and Future Directions

Our findings highlight the potential for ERP markers of habituation and novelty detection measured before six months of age to provide insight into later imitation abilities and memory development across settings. Such markers may thus be a useful addition to behavioural markers in identifying infants developing sub-optimally to provide early intervention.

## Figures and Tables

**Table 1:** Infant sample characteristics for infants included and excluded in the deferred imitation analysis at 12 months.

Infant Characteristics								
	Cohort Gambia				Cohort UK			
	Included		Excluded		Included		Excluded	
Sex (% female)	49.9		50		46.3		55	
	1 month		5 months		1 month		5 months	
	Included	Excluded	Included	Excluded	Included	Excluded	Included	Excluded
	X ± SD	X ± SD	X ± SD	X ± SD	X ± SD	X ± SD	X ± SD	X ± SD
Age (days)	42.22 ± 25.99	47.43 ± 32.51	159.51 ± 9.78	162.7 ± 12.85	32.77 ± 5.29	35.0 ± 6.74	155.74 ± 6.95	155.89 ± 5.77
Weight (kg)	4.23 ± 0.60	4.31 ± 0.54	6.84 ± 0.77	6.78 ± 0.89	4.35 ± 0.52	4.38 ± 0.58	7.19 ± 0.92	7.07 ± 0.85
Length (cm)	53.01 ± 2.08	53.08 ± 1.86	64.07 ± 2.06	64.21 ± 2.76	53.93 ± 2.17	54. 17 ± 1.93	64.42 ± 2.19	64.56 ± 2.32
Head circumference (cm)	36.58 ± 1.17	36.74 ± 1.19	41.26 ± 1.29	41.31 ± 1.46	37.34 ± 1.21	37.96 ± 1.02	42.84 ± 1.24	43.26 ± 1.15
Weight-for-age	−0.55 ± 0.95	−0.43 ± 0.85	−0.61 ± 0.95	−0.75 ± 1.09	−0.14 ± 0.81	−0.25 ± 1.02	−0.16 ± 1.12	−0.18 ± 0.95
Length-for-age	−0.93 ± 0.96	−0.56 ± 0.86	−0.60 ± 0.92	−0.631 ± 1.92	−0.30 ± 1.07	−0.32 ± 0.97	−0.38 ± 0.97	−0.23 ± 1.13
Head circumference for age	−0.59 ± 0.88	−0.47 ± 0.944	−0.74 ± 0.91	−0.78 ± 0.99	0.57 ± 0.90	0.67 ± 0.72	0.56 ± 0.94	0.99 ± 0.69
Weight-for-length	0.40 ± 1.08	0.14 ± 1.05	−0.23 ± 1.06	−0.37 ± 0.96	0.15 ± 1.06	−0.28 ± 1.09	0.17 ± 1.22	0.04 ± 0.95
Mullen Scales of Early Learning—VDQ	*not collected*	*not collected*	12.45 ± 2.71	12.91 ± 1.95	*not collected*	*not collected*	11.16 ± 2.09	11.41 ± 1.37
Mullen Scales of Early Learning—PDQ	*not collected*	*not collected*	14.74 ± 2.79	14.7 ± 2.245	*not collected*	*not collected*	14.17 ± 2.43	14.87 ± 1.59

Note. No group-differences were found on the above indicators after FDR correction for multiple comparisons. VDQ = verbal development quotient, PDQ = non-verbal development quotient.

**Table 2:** Maternal sample characteristics for infants included and excluded in the deferred imitation analysis at 12 months.

Maternal Characteristics at Birth				
	Cohort Gambia		Cohort UK	
	Included	Excluded	Included	Excluded
Maternal age	29.29 ± 6.55	29.95 ± 6.723	33.07 ± 2.99	32.81 ± 2.91
Parity	4.38 ± 2.39	4.38 ± 2.92	1.25 ± 0.49	1.45 ± 0.61
Gestational age	39.84 ± 1.93	39.42 ± 1.97	40.37 ± 1.29	39.98 ± 1.36

Note. No group-differences were found on the above indicators after FDR correction for multiple comparisons.

**Table 3:** Descriptive statistics for behavioural deferred imitation responses.

	Cohort Gambia		Cohort UK	
	8 Months	12 Months	8 Months	12 Months
*n*	144	182	29	41
	X ± SD	X ± SD	X ± SD	X ± SD
Baseline	1.500 ± 1.537	1.401 ± 1.390	2.621 ± 1.613	2.512 ± 1.583
Immediate	0.640 ± 0.910	0.890 ± 0.879	1.517 ± 1.242	2.244 ± 1.220
Deferred	1.217 ± 1.294	1.698 ± 1.202	3.462 ± 1.861	4.902 ± 2.615

## Data Availability

Data supporting this paper will be made available subject to established data sharing agreements.

## References

[1] Bandura A. (1969). Social-learning theory of identificatory processes. Handb. Social. Theory Res..

[2] Kawai M. (1965). Newly-acquired pre-cultural behavior of the natural troop of Japanese monkeys on Koshima Islet. Primates.

[3] Rumbaugh D.M., Savage-Rumbaugh E.S., Mackintosh N.J. (1994). Language in comparative perspective. Animal Learning and Cognition.

[4] Piaget J. (2013). Play, Dreams and Imitation in Childhood.

[5] De Haan M., Mishkin M., Baldeweg T., Vargha-Khadem F. (2006). Is deferred imitation a test of cognitive recall?. Trends Neurosci..

[6] Heimann M., Nordqvist E., Rudner M., Johansson M., Lindgren M. (2013). Associative learning measured with ERP predicts deferred imitation using a strict observation only design in 14 to 15 month old children. Scand. J. Psychol..

[7] Meltzoff A.N. (1985). Immediate and deferred imitation in fourteen-and twenty-four-month-old infants. Child Dev..

[8] Meltzoff A.N. (1988). Infant imitation and memory: Nine-month-olds in immediate and deferred tests. Child Dev..

[9] Meltzoff A.N., Moore M.K. (1983). Newborn infants imitate adult facial gestures. Child Dev..

[10] Jones E.J., Herbert J.S. (2006). Exploring memory in infancy: Deferred imitation and the development of declarative memory. Infant Child Dev. Int. J. Res. Pract..

[11] Barr R., Dowden A., Hayne H. (1996). Developmental changes in deferred imitation by 6-to 24-month-old infants. Infant Behav. Dev..

[12] Carver L.J., Bauer P.J., Nelson C.A. (2000). Associations between infant brain activity and recall memory. Dev. Sci..

[13] Bauer P.J., Wenner J., Dropik P.L., Wewerka S.S. (2000). Parameters of remembering and forgetting in the transition from infancy to early childhood. Monogr. Soc. Res. Child Dev..

[14] Hayne H., Boniface J., Barr R. (2000). The development of declarative memory in human infants: Age-related changes in deffered imitation. Behav. Neurosci..

[15] Learmonth A.E., Lamberth R., Rovee-Collier C. (2004). Generalization of deferred imitation during the first year of life. J. Exp. Child Psychol..

[16] Klein P.J., Meltzoff A.N. (1999). Long-term memory, forgetting, and deferred imitation in 12-month-old infants. Dev. Sci..

[17] Meltzoff A. (1993). The role of imitation in understanding persons and developing theory of mind. Understanding Other Minds: Perspectives from Autism.

[18] Barr R., Hayne H. (1996). The effect of event structure on imitation in infancy: Practice makes perfect?. Infant Behav. Dev..

[19] Hayne H., MacDonald S., Barr R. (1997). Developmental changes in the specificity of memory over the second year of life. Infant Behav. Dev..

[20] Fivush R., Kuebli J., Clubb P.A. (1992). The structure of events and event representations: A developmental analysis. Child Dev..

[21] Hudson J., Nelson K. (1986). Repeated encounters of a similar kind: Effects of familiarity on children’s autobiographic memory. Cogn. Dev..

[22] Murachver T., Pipe M.E., Gordon R., Owens J.L. Hooked on scripts: Generalized event memories acquired through direct experience and stories. Proceedings of the Biennial Meeting of the Society for Research in Child Development.

[23] Bauer P.J., Hertsgaard L.A., Wewerka S.S. (1995). Effects of experience and reminding on long-term recall in infancy: Remembering not to forget. J. Exp. Child Psychol..

[24] Bauer P.J., Mandler J.M. (1989). One thing follows another: Effects of temporal structure on 1-to 2-year-olds’ recall of events. Dev. Psychol..

[25] Bauer P.J., Shore C.M. (1987). Making a memorable event: Effects of familiarity and organization on young children’s recall of action sequences. Cogn. Dev..

[26] Eckerman C.O., Whitehead H. (1999). How toddler peers generate coordinated action: A cross-cultural exploration. Early Educ. Dev..

[27] Nielsen M., Tomaselli K. (2010). Overimitation in Kalahari Bushman children and the origins of human cultural cognition. Psychol. Sci..

[28] Goertz C., Lamm B., Graf F., Kolling T., Knopf M., Keller H. (2011). Deferred imitation in 6-month-old German and Cameroonian Nso infants. J. Cogn. Educ. Psychol..

[29] Graf F., Borchert S., Lamm B., Goertz C., Kolling T., Fassbender I., Teubert M., Vierhaus M., Freitag C., Spangler S. (2014). Imitative learning of Nso and German infants at 6 and 9 months of age: Evidence for a cross-cultural learning tool. J. Cross Cult. Psychol..

[30] Teiser J., Lamm B., Boning M., Graf F., Gudi H., Goertz C., Fassbender I., Freitag C., Spangler S., Teubert M. (2014). Deferred imitation in 9-month-olds: How do model and task characteristics matter across cultures?. Int. J. Behav. Dev..

[31] Correa-Chávez M., Rogoff B. (2009). Children’s attention to interactions directed to others: Guatemalan mayan and european american patterns. Dev. Psychol..

[32] Wheeler A.C., Gwaltney A., Raspa M., Okoniewski K.C., Berry-Kravis E., Botteron K.N., Budimirovic D., Hazlett H.C., Hessl D., Losh M. (2021). Emergence of developmental delay in infants and toddlers with an FMR1 mutation. Pediatrics.

[33] Yaari M., Mankuta D., Gadassi A.H., Friedlander E., Bar-Oz B., Eventov-Friedman S., Maniv N., Zucker D., Yirmiya N. (2018). Early developmental trajectories of preterm infants. Res. Dev. Disabil..

[34] Bauer P.J., Wiebe S.A., Carver L.J., Waters J.M., Nelson C.A. (2003). Developments in long-term explicit memory late in the first year of life: Behavioral and electrophysiological indices. Psychol. Sci..

[35] Bauer P.J., Wiebe S.A., Carver L.J., Lukowski A.F., Haight J.C., Waters J.M., Nelson C.A. (2006). Electrophysiological indexes of encoding and behavioral indexes of recall: Examining relations and developmental change late in the first year of life. Dev. Neuropsychol..

[36] Morasch K.C., Bell M.A. (2009). Patterns of brain-electrical activity during declarative memory performance in 10-month-old infants. Brain Cogn..

[37] Nordqvist E., Rudner M., Johansson M., Lindgren M., Heimann M. (2015). The relationship between deferred imitation, associative memory, and communication in 14-months-old children. Behavioral and electrophysiological indices. Front. Psychol..

[38] Katus L., Mason L., Milosavljevic B., McCann S., Rozhko M., Moore S.E., Elwell C.E., Lloyd-Fox S., de Haan M., Drammeh S. (2020). ERP markers are associated with neurodevelopmental outcomes in 1–5 month old infants in rural Africa and the UK. NeuroImage.

[39] Lloyd-Fox S., Blasi A., McCann S., Rozhko M., Katus L., Mason L., Austin T., Moore S.E., Elwell C.E., The BRIGHT Project Team (2019). Habituation and novelty detection fNIRS brain responses in 5-and 8-month-old infants: The Gambia and UK. Dev. Sci..

[40] Lunn P.G., Northrop-Clewes C.A., Downes R.M. (1991). Intestinal permeability, mucosal injury, and growth faltering in Gambian infants. Lancet.

[41] Kushnerenko E., Winkler I., Horváth J., Näätänen R., Pavlov I., Fellman V., Huotilainen M. (2007). Processing acoustic change and novelty in newborn infants. Eur. J. Neurosci..

[42] Milosavljevic B., Vellekoop P., Maris H., Halliday D., Drammeh S., Sanyang L., Darboe M.K., Elwell C., Moore S.E., Lloyd-Fox S. (2019). Adaptation of the Mullen Scales of Early Learning for use among infants aged 5-to 24-months in rural Gambia. Dev. Sci..

[43] Mullen E.M. (1995). Mullen Scales of Early Learning.

[44] Delehanty A.D., Stronach S., Guthrie W., Slate E., Wetherby A.M. (2018). Verbal and nonverbal outcomes of toddlers with and without autism spectrum disorder, language delay, and global developmental delay. Autism Dev. Lang. Impair..

[45] Jeffreys H. (1961). Theory of Probability.

[46] Carver L.J., Bauer P.J. (2001). The dawning of a past: The emergence of long-term explicit memory in infancy. J. Exp. Psychol. Gen..

[47] Otte R., Winkler I., Braeken M., Stekelenburg J., van der Stelt O., Bergh B.R.V.D. (2013). Detecting violations of temporal regularities in waking and sleeping two-month-old infants. Biol. Psychol..

[48] Van den Heuvel M.I., Otte R.A., Braeken M.A., Winkler I., Kushnerenko E., Bergh B.R.V.D. (2015). Differences between human auditory event-related potentials (AERPs) measured at 2 and 4 months after birth. Int. J. Psychophysiol..

[49] Adlam A.-L.R., Vargha-Khadem F., Mishkin M., de Haan M. (2005). Deferred imitation of action sequences in developmental amnesia. J. Cogn. Neurosci..

